# 

**Published:** 1916-09

**Authors:** 


PUBLISHED MONTHLY. SUBSCRIPTION $1.00

### ATLANTA JOURNAL-RECORD OF MEDICINE

Successor to { Atlanta Medical and Surgical Journal, Established 1855. and Southern Medical Record, Established 1870. } 63rd Year

Vol. LXIII ATLANTA, GA., SEPTEMBER, 1916 No. 6

